# NAD^+^, Axonal Maintenance, and Neurological Disease

**DOI:** 10.1089/ars.2023.0350

**Published:** 2023-12-05

**Authors:** Athanasios S. Alexandris, Vassilis E. Koliatsos

**Affiliations:** ^1^ Department of Pathology, Johns Hopkins University School of Medicine, Baltimore, Maryland, USA.; ^2^ Department of Neurology, and Johns Hopkins University School of Medicine, Baltimore, Maryland, USA.; ^3^ Department of Psychiatry and Behavioral Sciences, Johns Hopkins University School of Medicine, Baltimore, Maryland, USA.

**Keywords:** SARM1, neurodegeneration, Wallerian degeneration, nicotinamide, axonal degeneration, pellagra

## Abstract

**Significance:**

The remarkable geometry of the axon exposes it to unique challenges for survival and maintenance. Axonal degeneration is a feature of peripheral neuropathies, glaucoma, and traumatic brain injury, and an early event in neurodegenerative diseases. Since the discovery of Wallerian degeneration (WD), a molecular program that hijacks nicotinamide adenine dinucleotide (NAD^+^) metabolism for axonal self-destruction, the complex roles of NAD^+^ in axonal viability and disease have become research priority.

**Recent Advances:**

The discoveries of the protective Wallerian degeneration slow (Wld^S^) and of sterile alpha and TIR motif containing 1 (SARM1) activation as the main instructive signal for WD have shed new light on the regulatory role of NAD^+^ in axonal degeneration in a growing number of neurological diseases. SARM1 has been characterized as a NAD^+^ hydrolase and sensor of NAD^+^ metabolism. The discovery of regulators of nicotinamide mononucleotide adenylyltransferase 2 (NMNAT2) proteostasis in axons, the allosteric regulation of SARM1 by NAD^+^ and NMN, and the existence of clinically relevant windows of action of these signals has opened new opportunities for therapeutic interventions, including SARM1 inhibitors and modulators of NAD^+^ metabolism.

**Critical Issues:**

Events upstream and downstream of SARM1 remain unclear. Furthermore, manipulating NAD^+^ metabolism, an overdetermined process crucial in cell survival, for preventing the degeneration of the injured axon may be difficult and potentially toxic.

**Future Directions:**

There is a need for clarification of the distinct roles of NAD^+^ metabolism in axonal maintenance as contrasted to WD. There is also a need to better understand the role of NAD^+^ metabolism in axonal endangerment in neuropathies, diseases of the white matter, and the early stages of neurodegenerative diseases of the central nervous system. *Antioxid. Redox Signal.* 39, 1167–1184.

## The Axon as a Relatively Autonomous Unit Within Neuron and the Role of Nicotinamide Adenine Dinucleotide

Even before Deiters first identified and named the axon the “axial cylindrical process” (Deiters and Guillery, 2013), Augusts Waller had described a phenomenon that would eventually change the understanding of axonal biology, that is, that the distal part of a transected nerve, after a quiescent period of several days, abruptly and entirely breaks down in what he described as “coagulation … into separate particles” (Waller, 1850).

Waller (1850) had proposed that these characteristic changes may underlie diseases of the peripheral nerves and the brain. Still, it would take >150 years to better understand the mechanism of this phenomenon, since dubbed Wallerian degeneration (WD), and its intimate association with the metabolism of nicotinamide adenine dinucleotide (oxidized) (NAD^+^), a promiscuous redox cofactor essential for a wide range of cellular functions.

According to the classical view, the axon is a highly differentiated process that contains large amounts of cytosol (axoplasm) and realizes the efferent electric impulse-based communication of the nerve cell with other neurons in the same circuit (Koliatsos and Alexandris, 2019; Nissl, 1894). At the dawn of modern molecular neurobiology, emphasis was shifted to the axon as the trophic conduit between the soma and the nerve terminal or the postsynaptic neuron, especially in the retrograde direction (Ehlers et al., 1995; Koliatsos and Price, 1996; Koliatsos and Price, 1993).

**FIG. 1.**
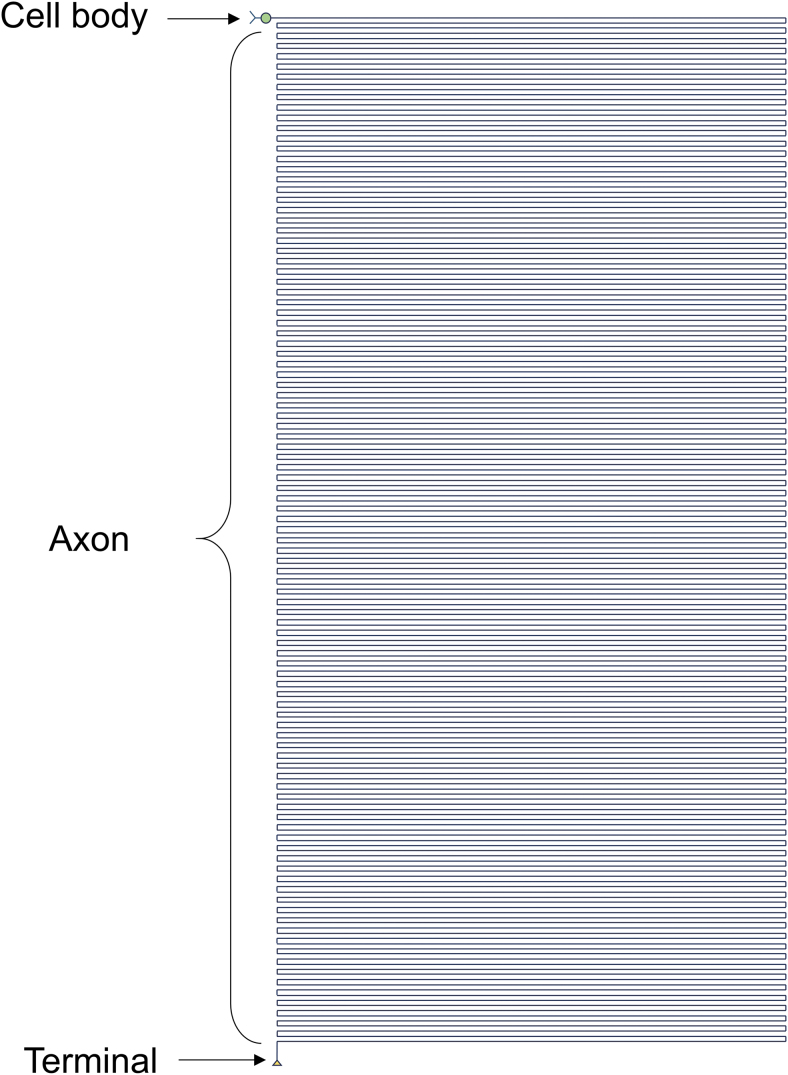
**The outstanding geometry of the axon.** This diagram shows the relative length of the axon of a human motor neuron drawn to scale with its cell body diameter. For a similar approach, see also Devor (1999) and Koliatsos and Alexandris (2019).

Even these basic facts, however, underestimate the consequences of a truly outstanding geometry, the detail of which is increasingly unraveled with the use of modern anatomical tools. For example, a human motor neuron with a maximal 100 μm-diameter soma has an axon that runs for ≥1 m (Fig. 1), while a single human basal forebrain cholinergic neuron with a soma of diameter of 30 μm has an estimated cumulative axon length that exceeds 100 m with all its branches (Wu et al., 2014). Based on other estimates, the human brain has ≥170,000 km of total axon length (Marner et al., 2003).

The substantial metabolic burden of the axon coupled with its decentralized geometry represents challenges that cannot be readily met with support by the parent cell body alone. Indeed, axons are endowed with autonomous regulatory processes such as local protein synthesis (Lin et al., 2021), and cooperate with oligodendrocytes or Schwann cells for protein synthesis and shuttling of metabolites (Court et al., 2008; Philips and Rothstein, 2017). Despite such support, the axon remains a vulnerable structure and may form the nidus of neuropathology for a wide range of disorders (Adalbert and Coleman, 2013; Dadon-Nachum et al., 2011; Tagliaferro and Burke, 2016), a fact underlying the need to pay attention to the axon for neuroprotection and therapeutics (Bodian, 1952; Koliatsos and Alexandris, 2019; Tagliaferro and Burke, 2016).

Perhaps the most remarkable manifestation of the uniqueness of axon in the cell biology of neuron is its distinct responses to injury and handling of prodegenerative signals. In this regard, a key development was the discovery of Ola mice featuring a surprisingly slow WD after sciatic axotomy (Lunn et al., 1989; Perry et al., 1991). In these mice, transected axons can survive and maintain their ability to transmit action potentials for weeks (Tsao et al., 1994). This extraordinary phenotype was named Wallerian degeneration slow (Wld^S^) and is the result of a spontaneous genetic rearrangement encoding Wld^S^, a chimeric protein formed *de novo* by the in-frame fusion of the N-terminus of Ube4b (a ubiquitin ligase) and the full sequence of the nicotinamide mononucleotide adenylyltransferase 1 (NMNAT1) (Mack et al., 2001). The discovery of Ola mice and Wld^S^ showed that axonal degeneration is not the passive response to the deprivation of key support from the perikaryon, but the dictate of a distinct genetic program. In further support of the axonal specificity of this program, Wld^S^ appears to have no effect on the neuronal cell body in classical models of apoptosis; that is, NGF deprivation of sympathetic neurons or the neonatal motor neuron degeneration after axotomy (Adalbert et al., 2006; Deckwerth and Johnson, 1994). Conversely, robust antiapoptotic strategies such as Bak or Bax deletion and Bcl-2 overexpression, protect the soma but do not prevent WD (Burne et al., 1996; Whitmore et al., 2003).

The core component of Wld^S^, NMNAT1, is responsible for the final enzymatic step in the synthesis of NAD^+^. It is therefore no surprise that, since the discovery of Wld^S^, research on NAD^+^ metabolism has become central in our effort to understand axonal viability and degeneration. In this review, we focus on the axon as a neuronal domain that is functionally and metabolically distinct from the cell body, and discuss the current understanding of axonal NAD^+^ metabolism in health and disease. To propose unifying concepts useful for further research, we often combine evidence from multiple models (*e.g*., cell-free, *in vitro* and *in vivo* conditions), with focus on mammalian systems.

## Essentials of NAD^+^ Metabolism with Emphasis on Mammalian Neurons

**FIG. 2.**
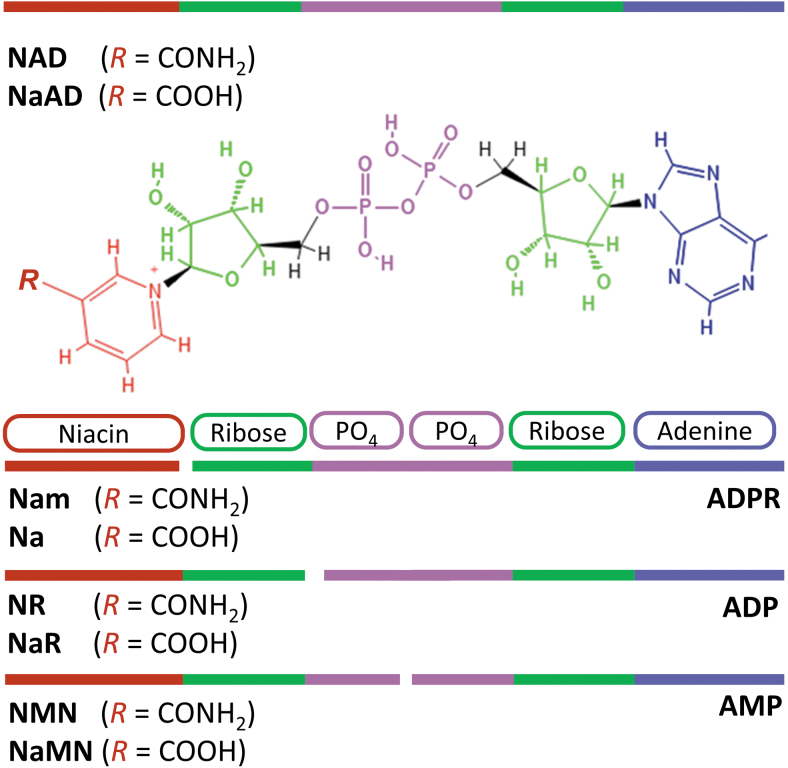
**Chemical structure of NAD^+^ and related metabolites.** Niacin (*red*) typically refers to Na and its amide, Nam. Biosynthetic pathways can utilize either form to create amidated and nonamidated derivatives. Phosphoribosylation of niacin produces NaMN and NMN, and the further addition of an adenosine monophosphate group gives rise to NaAD and NAD. Dephosphorylation of NaMN or NMN can generate NaR and NR, respectively. For mammalian biosynthetic pathways, see Figure 3. Na, nicotinic acid; NaAD, nicotinic acid adenine dinucleotide; NAD^+^, nicotinamide adenine dinucleotide (oxidized); Nam, nicotinamide; NaMN, nicotinic acid mononucleotide; NaR, nicotinic acid riboside; NMN, nicotinamide mononucleotide; NR, nicotinamide riboside.

Nicotinamide adenine dinucleotide (Fig. 2) is a coenzyme found in all living cells and is essential for a wide range of biological processes. It was first identified as a factor that increases the rate of fermentation in yeast extracts and subsequently as the major hydride acceptor in reduction–oxidation (“redox”) reactions, including glycolysis, the Krebs cycle, and fatty acid oxidation, in all of which NAD^+^ is reduced to NADH (Bogan and Brenner, 2008). NADH is the main hydrogen donor in ATP synthesis through the mitochondrial electron transport chain (ETC) (Bogan and Brenner, 2008). The phosphorylated form of NAD(H), NADP(H), which is synthesized by NAD kinase, is involved in other important physiological processes such as the biosynthesis of fatty acids, phospholipids, and amino acids, and in antioxidant defense (Agledal et al., 2010).

Over the last decades, NAD^+^ has emerged as a substrate for enzymes that play important roles in diverse functions beyond redox metabolism, such as regulation of gene expression, DNA repair, mitochondrial function, autophagy, inflammation, and apoptosis (Bai, 2015; Wu et al., 2022). NAD^+^-consuming enzymes break down NAD^+^ into nicotinamide (Nam) and ADP-ribosyl products. The latter includes the family of sirtuins, which are NAD^+^-dependent deacylases (Feldman et al., 2012), the ADPribosyltransferases (ARTs), and poly-ADPribosylatransferases (PARPs), which post-translationally modify proteins with monomers or polymers of ADPribose (Bai, 2015; Challa et al., 2021), and the family of NAD^+^ hydrolases such as CD157, CD73, and CD38, which produce free or cyclical ADP-ribose (ADPR, cADPR) (Gasparrini et al., 2021). The activity of NAD^+^-consuming enzymes is regulated by NAD^+^ availability and feedback inhibition by NAD^+^ precursors. Conversely, by degrading NAD^+^, NAD^+^-consuming enzymes can regulate local NAD^+^ availability, and subsequently impact both redox and nonredox functions of NAD^+^.

**FIG. 3.**
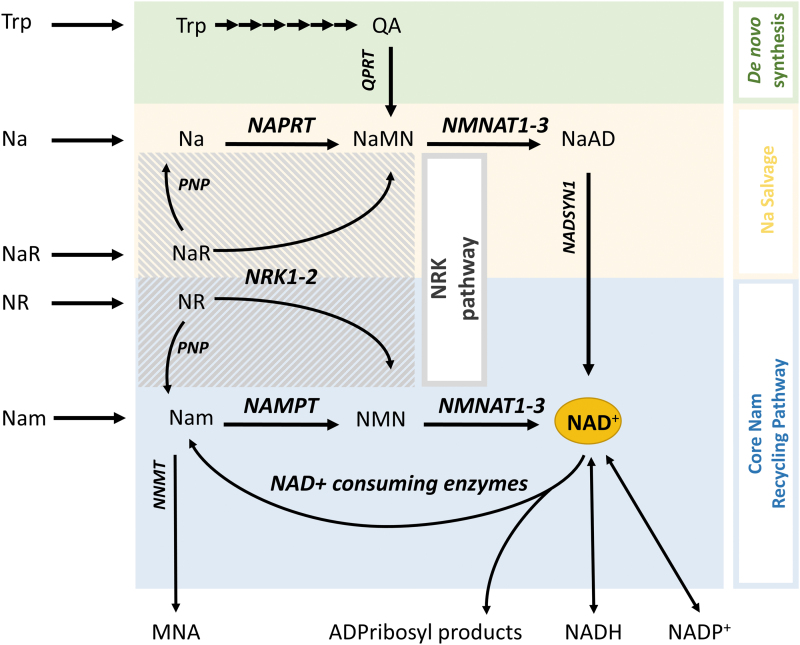
**NAD^+^ biosynthetic pathways in mammalian cells.** There are three main NAD^+^ biosynthetic pathways, each starting with a different NAD^+^ precursor. The core Nam recycling pathway (*blue box*) utilizes Nam produced from intracellular NAD^+^ degradation or imported from the extracellular space. Nam is converted to NMN by NAMPT, and this is in turn converted to NAD^+^ by any of the rate-limiting isoforms of the NMN adenylyltransferase enzymes (NMNAT1–3). In a manner similar to the Nam recycling pathway, the Na salvage pathway (also known as Preiss–Handler pathway; *yellow box*) converts Na to NaMN by NAPRT, and NaMN to NaAD by NMNAT. NaAD is then converted to NAD^+^ by NADSYN. In the *de novo* synthesis pathway (*green box*), tryptophan is converted to QA, which is then utilized for NAD^+^ synthesis *via* NaMN and NaAD intermediates. Extracellular NaR and NR can also be imported and utilized intracellularly after phosphorylation by NRK 1 or 2, generating NaMN or NMN, respectively (*gray striped box*). NAD^+^ exists in equilibrium with its reduced and phosphorylated forms, and can be degraded by different NAD^+^-consuming enzymes (see Essentials of NAD^+^ Metabolism). While most of intracellular Nam is recycled, it can also be eliminated and excreted after its methylation by NNMT to form MNA. MNA, 1-methylnicotinamide; NADSYN, NAD^+^ synthase; NAMPT, nicotinamide phosphoribosyltransferase; NAPRT, nicotinate phosphoribosyltransferase; NMNAT, nicotinamide mononucleotide adenylyltransferase; NNMT, nicotinamide n-methyltransferase; NRK, nicotinamide riboside kinase; PNP, purine nucleoside phosphorylase; QA, quinolinic acid; QPRT, quinolinate phosphoribosyl transferase.

To maintain NAD^+^ levels, cells employ several discrete, evolutionary conserved, biosynthetic pathways (Yang and Sauve, 2016) (Fig. 3). In mammals, these include the *de novo synthesis pathway* (also known as the kynurenine pathway), which synthesizes NAD^+^ from tryptophan, and two salvage pathways that generate NAD^+^ from niacin (vitamin B3). Niacin exists in two main forms, nicotinic acid (Na) that is converted to NAD^+^ *via* the *Na salvage* (“Preiss–Handler”) *pathway* and niacin amide (Nam), converted to NAD^+^ *via* the core *Nam recycling pathway*. The Nam recycling pathway involves two steps: first is the formation of nicotinamide mononucleotide (NMN) by Nam phosphoribosyltransferase (NAMPT); and second is the synthesis of NAD^+^ from NMN by NMNAT (Fig. 3). NMNAT occurs in three isoforms: NMNAT1 that is expressed in the nucleus, NMNAT2 that is expressed in the cytosol including axons, and NMNAT3 that is mostly expressed in mitochondria (Fortunato et al., 2022).

Most cells, including neurons, do not express the enzymatic machinery to convert tryptophan or dietary Na to NAD^+^, and rely mostly on recycling Nam produced from intracellular NAD^+^ degradation (Liu et al., 2018b). This is a very efficient process, and it is estimated that the entire NAD^+^ pool is being regenerated 2–4 times per day (Yang and Sauve, 2016). However, intracellular recycling of NAD^+^ is not unlimited, because Nam is also eliminated by conversion to 1-methylnicotinamide (MNA) *via* the action of cytosolic Nam n-methyltransferase (NNMT) and then urinary excretion (Pissios, 2017) (Fig. 3). Therefore, most cells (including neurons) rely on extracellular Nam and other NAD^+^ precursors released in the circulation by the liver (synthesized *de novo*) or the intestine (from the diet) (Liu et al., 2018b). *De novo* synthesis is not as efficient and is energetically costly (Croft et al., 2020), and dietary niacin is necessary to prevent NAD^+^ deficiency, especially in humans (Feuz et al., 2023).

**FIG. 4.**
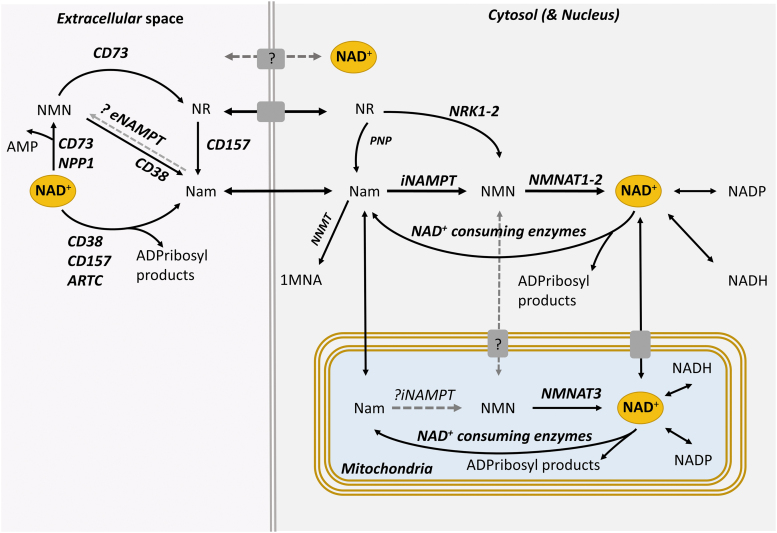
**Regulation of NAD^+^ pools across subcellular compartments.** In axons, the core Nam recycling pathway is the main source of NAD^+^. NAD^+^ and NMN do not cross cellular membranes readily, and form distinct subcellular pools. The specifics of mitochondrial NAD^+^ biogenesis are debated, but NAD^+^ may be imported/exported *via* putative NAD^+^ transporters, while NAD^+^ might be also generated from imported NMN or mitochondrial Nam. Extracellular NAD^+^ precursors and NAD^+^ are further regulated by ectoenzymes such as CD 38, CD73, and CD157. While NAD^+^ levels are independently regulated in different subcellular compartments, NAD^+^ pools may affect each other *via* effects on NAD^+^ precursors or possibly *via* NAD^+^ transporters. Based on Cambronne and Kraus (2020) and Gasparrini et al. (2021). ARTC, ADP-ribosyltransferase C2/C3 toxin-like; eNAMPT, extracellular nicotinamide phosphoribosyltransferase; iNAMPT, intracellular nicotinamide phosphoribosyltransferase; NPP1, nucleotide pyrophosphatase/phosphodiesterase 1.

In addition to the three main pathways reviewed, two paragraphs above, cells can synthesize NAD^+^ from Na riboside (NaR) and Nam riboside (NR) that represent a third form of vitamin B3 (Bieganowski and Brenner, 2004). NaR and NR can be phosphorylated by Nam riboside kinases 1 and 2 (NRK1–2) to produce nicotinic acid mononucleotide (NaMN) and NMN that are then converted to NAD^+^ in the Na salvage and Nam recycling pathways, respectively (Bieganowski and Brenner, 2004) (Fig. 3). Although the NRK pathway is not essential for intracellular NAD^+^ salvage, it is indispensable for the utilization of extracellular NAD^+^ precursors such as NR and the membrane-impermeable NMN (Ratajczak et al., 2016) (Fig. 4). Notably, the expression of NRK2 can be increased by >20-fold after axotomy in neurons (Sasaki et al., 2006), indicating a possible supporting role of this pathway in NAD^+^ synthesis under conditions of axonal injury.

Although aspects of NAD^+^ metabolism are conserved across species and tissues, the synthesis and degradation of NAD^+^ are differentially regulated among organs, cell types, and subcellular spaces, and also depend on timing, conditions painting a spatial and temporal landscape of high complexity. At the organism level, various organs have different preferences for specific NAD^+^ precursors and show major differences in NAD^+^ turnover (Liu et al., 2018b). At the cellular level, NAD^+^ metabolism is highly partitioned, and different compartments have distinct NAD^+^ pools (Cambronne and Kraus, 2020) (Fig. 4). This is in part because each membrane-bound cell domain including the cytosol, nucleus, mitochondria, and vesicles utilizes different NAD^+^-biosynthetic and NAD^+^-degrading enzymes, and NAD^+^ itself has limited diffusion through membranes (Cambronne and Kraus, 2020). Although such compartmentalization provides cells with the ability to protect specific pools of NAD^+^, the latter may overcome membrane barriers by using NAD^+^ transporters. This is especially relevant under extreme conditions; for example, depletion of NAD^+^ in the cytosol may eventually empty NAD^+^ stores in mitochondria (Cambronne and Kraus, 2020).

## NAD^+^ Metabolism as the Gatekeeper of Axonal Viability: From Pellagra to WD

**FIG. 5.**
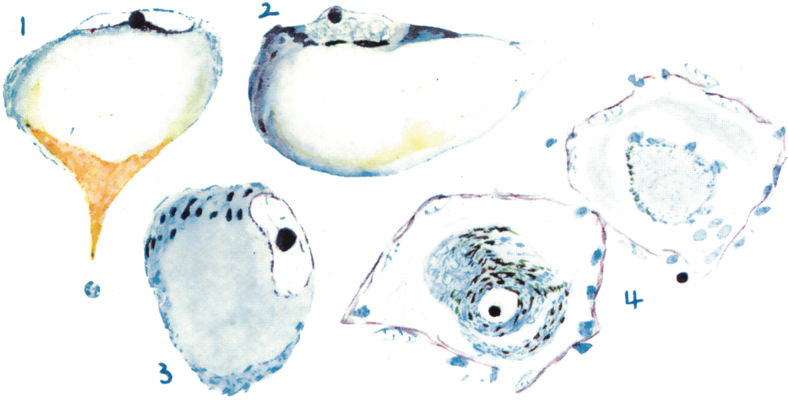
**Axon reaction (chromatolysis) in pellagra.** Nissl-stained neurons from patients with pellagrous encephalopathy are reproduced from Singer and Pollock (1913). Cells 1 and 2 are from cerebral cortex, cell 3 is from the ventral horn, and cell 4 is from a sympathetic ganglion. Axon reaction is a classical marker of axonal injury.

The importance of NAD^+^ metabolism for neuronal and axonal viability was classically demonstrated with the discovery that a diet deficient in niacin causes pellagra, a syndrome characterized by light-sensitive dermatitis (*pelle agra* is Italian for rough skin), diarrhea and, importantly, dementia. The neuropathology of pellagrous encephalopathy has been poorly studied because of its near disappearance due to improved diet in most countries (Leigh, 1952). However, in an early series of 14 cases, Singer and Pollock (1913) had found widespread axonal degeneration throughout the nervous system and chromatolysis, a marker of axonal injury, in large pyramidal neurons in cortex as well as in neurons of the hippocampus, cerebellum, subcortical gray matter, and cranial nerve nuclei (Fig. 5).

Although pellagra from niacin-poor diet is nowadays rare, pellagra has also been associated with malnutrition or malabsorption due to anorexia nervosa, alcoholism, and Crohn's disease, and also with medications that interfere with NAD^+^ biosynthesis such as isoniazid (Feuz et al., 2023); neuropathological findings in these cases are similar to the ones encountered in dietary pellagra (Ishii and Nishihara, 1985). There is also a rare genetic form of pellagra, Hartnup disease, caused by autosomal recessive mutations in *SLC6A19* that also results in niacin deficiency because of impaired absorption and retention of neutral amino acids, including the niacin precursor tryptophan. Hartnup disease is characterized by episodic cutaneous and neurological symptoms and, in a manner analogous to dietary and digestive pellagra, features neuronal loss and axonal degeneration in various sites in the brain (Schmidtke et al., 1992; Tahmoush et al., 1976). While the mechanism of axonal vulnerability and degeneration in pellagra remains unknown, the disease and its causes bring into focus the role of NAD^+^ dysmetabolism in neurodegeneration and the vulnerability of the axon under these conditions.

**FIG. 6.**
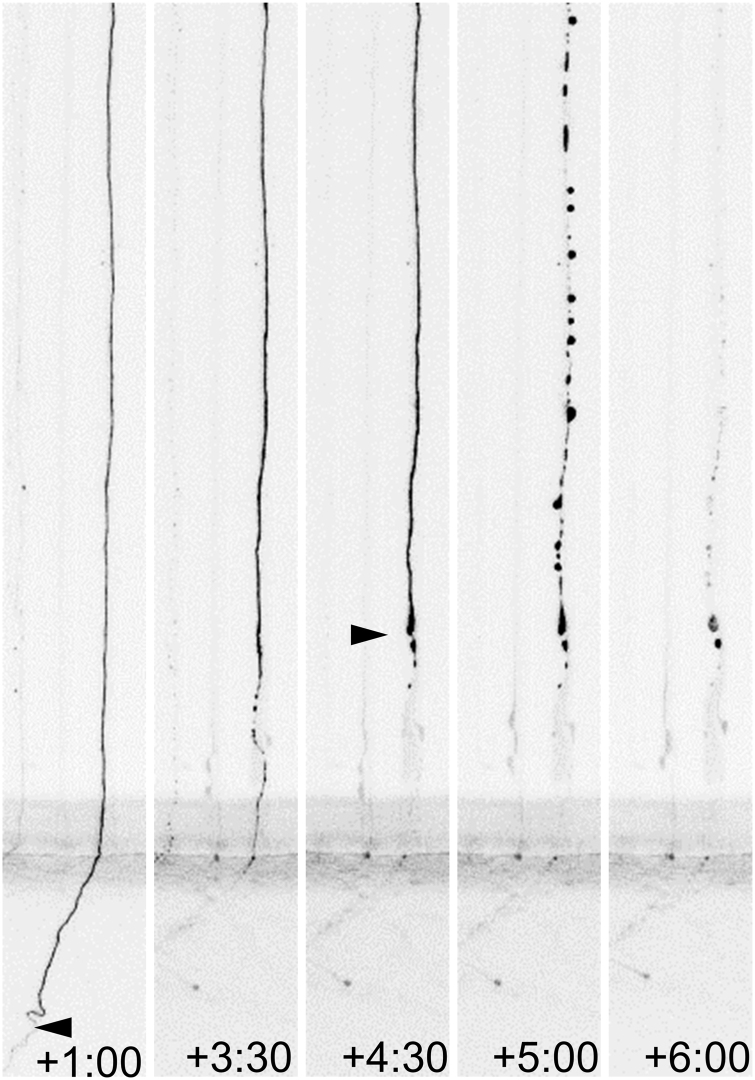
**WD after axotomy.** A transected axon of a mouse DRG neuron is shown at different time points after injury (h:mm). Primary mouse DRG neurons were obtained from YFP-16 mice and cultured in microfluidic devices for separation of cell bodies (not shown; toward the bottom of the field of view) and axons. Axotomy was performed by a razor blade and axon segments distal to the injury were visualized based on their YFP fluorescence. The distal stump shows limited retraction in the first 4.5 h (*arrowheads*), before a lengthwide fragmentation and subsequent dissolution at 5–6 h. DRG, dorsal root ganglion; WD, Wallerian degeneration.

As indicated in the beginning of this review, WD is classically referred to as the stereotypical fragmentation and dissolution of the distal segment of the axon after axotomy (Fig. 6), although, as we shall discuss in the following sections, it may also result from perturbations such as arrest of axonal transport, neurotoxic damage to the axoskeleton, and oxidative stress/mitochondrial dysfunction.

The depletion of NMNAT2 shared among these diverse types of injury is the necessary condition for activation of the WD program: the replacement of NMNAT2 with the more stable Wld^S^ (Gilley and Coleman, 2010), overexpression of other axon-directed cytosolic constructs of NMNAT1 (Babetto et al., 2010; Sasaki et al., 2009), or exogenous transfer of NMNAT2 (Sasaki and Milbrandt, 2010), all suffice to suppress axonal fragmentation. NMNAT2 is labile, having a half-life of ∼40 min, and is rapidly depleted after injury; that is, within 4 h after axotomy *in vitro* (Gilley and Coleman, 2010; Milde et al., 2013). Its continuous replenishment by axon transport (Gilley and Coleman, 2010) and probably by local protein synthesis (Shigeoka et al., 2016) is central to maintenance of axonal viability; for example, knockdown of NMNAT2 with siRNAs leads to spontaneous axonal degeneration (Gilley and Coleman, 2010).

The requirement of NMNAT2 for axonal growth and maintenance is demonstrated by the fact that neurons deficient of NMNAT2 develop very short neurites, while knock out of *Nmnat2* in mice is perinatally lethal from lack of innervation of the diaphragm (Gilley et al., 2013). Mice with very low levels of *Nmnat2* expression survive, but develop significant axonopathy later in life (Gilley et al., 2019). The clinical significance of these findings is illustrated in two rare syndromes: fetal akinesia deformation sequence associated with loss-of-function mutation in *Nmnat2* (Lukacs et al., 2019) and two cases of childhood-onset polyneuropathy with erythromelalgia associated with a homozygous *Nmnat2* mutation resulting in reduced NMNAT2 activity (Huppke et al., 2019).

While NMNAT2 is required for axonal development and survival, and its loss is a trigger of WD, the mechanism by which loss of NMNAT activity leads to degeneration has been debated owing to several puzzling observations (Conforti et al., 2014). For instance, while the stimulation of NAD^+^ biosynthesis *before* axotomy delays axonal degeneration (Sasaki et al., 2006), the blockade of NAMPT, that is, the enzyme catalyzing the rate-limiting step in NAD biosynthesis (Fig. 3), has a paradoxical protective effect after axotomy (Alexandris et al., 2022; Di Stefano et al., 2015). Similarly, the expression of cytosolic NMNAT1 is robustly protective against axotomy even when NAMPT is inhibited and NAD^+^ biosynthesis is suppressed, a condition suggesting that NMNAT activity is more important than NAD^+^ levels (Sasaki et al., 2009; Shen et al., 2013). These paradoxes were partially reconciled by findings suggesting that the NMNAT substrate NMN is neurotoxic. First, loss of NMNAT activity in axons after injury leads to accumulation of NMN and clearance of that NMN by a bacterial NMN-deamidase can significantly delay axon degeneration despite loss of NAD^+^ after injury. Second, the protective effect of NAMPT inhibition can be reversed by exogenous NMN (Di Stefano et al., 2017), and this is especially striking when delayed for a few hours (Alexandris et al., 2022). This time-dependent effect is explained by the kinetics of NMNAT2 depletion: inhibiting NAMPT after loss of NMNAT activity reduces NMN without further decreasing NAD^+^ levels; and the addition of NMN is most toxic after the loss of NMNAT activity, that is, at a point at which it can no longer contribute to NAD^+^ synthesis (Alexandris et al., 2022).

The previous observations indicate that both NMN and NAD^+^ regulate WD. However, the mechanism by which they influence the activation of WD did not become apparent until the discovery and characterization of sterile alpha and TIR motif containing 1 (SARM1) as a novel NAD^+^ hydrolase, whose activity is necessary and is thought to be sufficient for triggering WD (Essuman et al., 2017; Osterloh et al., 2012).

## SARM1: NAD^+^ Hydrolase and Sensor of the State of NAD^+^ Metabolism

SARM1 is a 690 amino acid multidomain protein that is highly conserved from *Caenorhabditis elegans* to human (Mink et al., 2001). It contains a Toll-interleukin-1 receptor (TIR) domain, which has NAD^+^ glycohydrolase activity upon dimerization, two sterile alpha motif (SAM) domains, which facilitate its multimerization, an autoinhibitory armadillo repeat (ARM) domain, and a mitochondrial localization sequence (MLS) (Gerdts et al., 2015; Gerdts et al., 2013).

SARM1 was originally annotated as the fifth member of the myeloid differentiation primary response gene 88 (MyD88) family of mammalian proteins that are distinguished by their C-terminal TIR domain and are thought to act as immune adaptor proteins for Toll-like receptor signaling (Carty et al., 2006; Kim et al., 2007). However, the unexpected discovery of its NADase activity (Essuman et al., 2017) that is unique among MyD88 proteins places SARM1 within a larger, diverse family of TIR-domain proteins with important roles in innate immunity, from bacteria and plants to humans (DiAntonio et al., 2021). The intrinsic NADase activity of these proteins serves as a defense mechanism against pathogens by depleting intracellular NAD^+^ levels and causing cell death (DiAntonio et al., 2021; Wan et al., 2019).

The central role of SARM1 in WD was first identified on a *Drosophila melanogaster* screen for mutants with suppressed WD after axonal transection (Osterloh et al., 2012). In confirmatory experiments in mice, *Sarm1* knock out (KO) was shown to suppress classical WD in a manner similar to Wld^S^ (Gerdts et al., 2013; Osterloh et al., 2012), and further work over the last decade characterized the function of SARM1 as a NADase that is required for axon degeneration after injury and whose forced activation is sufficient to induce axonal degeneration in healthy neurons (Gerdts et al., 2015).

**FIG. 7.**
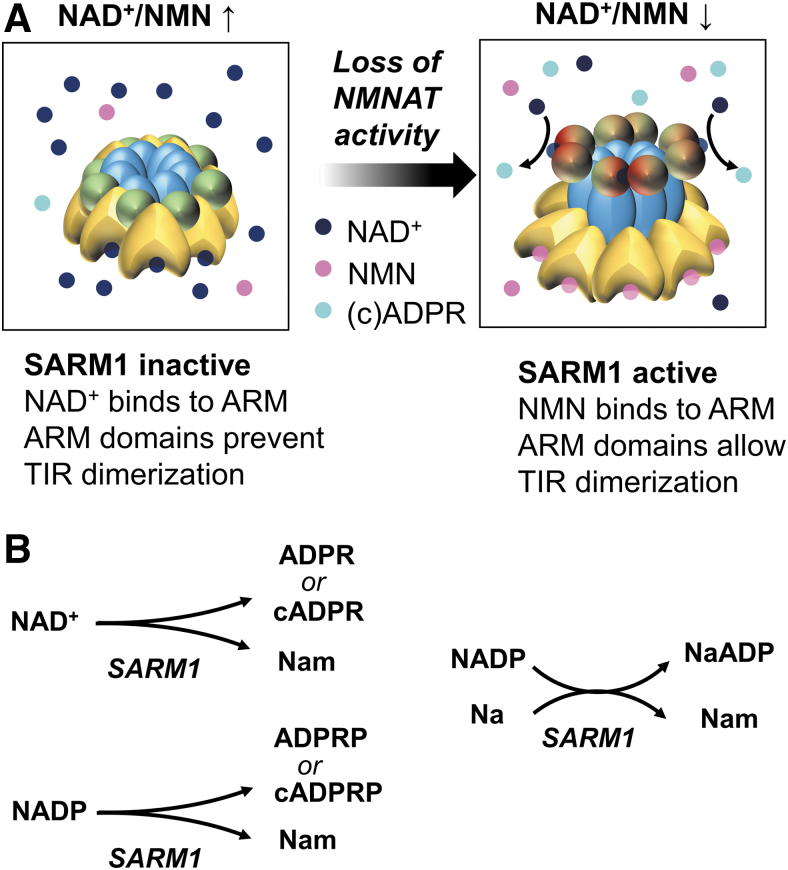
**A model of SARM1 activation. (A)** SARM1 is a multidomain protein that forms an octamer. SARM1 has a mitochondrial localization sequence (not shown), an ARM domain (*yellow*) that contains sites for allosteric binding of NAD^+^ and NMN, two SAM domains (*blue*), which facilitate multimerization, and a TIR domain with NAD(P)^+^ glycohydrolase activity (*green*). The SAM domains are assembled in an inner ring, while the ARM and TIR domains radiate outward. NAD^+^ and NMN compete for binding at allosteric sites in the ARM domain. NAD^+^ binding stabilizes ARM-TIR and ARM-SAM domain interactions, and prevents TIR dimerization. NMN binding induces a conformational change that allows TIR dimerization and enzymatic activation. **(B)** Types of reactions catalyzed by SARM1. Based on Icso and Thompson (2022). ARM, armadillo repeat; SAM, sterile alpha motif; SARM1, sterile alpha and TIR motif containing 1; TIR, toll-interleukin-1 receptor.

SARM1 is thought to form an octamer whose conformation is determined by competitive binding of NAD^+^, NMN, and related metabolites on an allosteric site within its ARM domain. Binding of NAD^+^ promotes an enzymatically inactive conformation, while binding of NMN facilitates the dimerization of the TIR domains and enables their glycohydrolase activity (Angeletti et al., 2022; Bratkowski et al., 2020; Figley et al., 2021; Jiang et al., 2020; Shen et al., 2021; Sporny et al., 2020). Based on findings in cell-free assays of purified human SARM1 and with pharmacological or genetic manipulations in neurons *in vitro*, the current model couples SARM1 activation to the NAD^+^/NMN ratio (Alexandris et al., 2022; Figley et al., 2021) and therefore, indirectly, to NMNAT activity (Fig. 7). In support of this model, we have shown that levels of cADPR, the main SARM1 product in neurons (Sasaki et al., 2020), correlate best with the NAD^+^/NMN ratio and not with the absolute levels of NAD^+^, NMN, or other metabolites in naïve or injured mammalian axons (Alexandris et al., 2022).

The deamidated NAD^+^ precursors NaR and NaMN have also been suggested to allosterically inhibit purified SARM1 (Angeletti et al., 2022; Sasaki et al., 2021), but the physiological significance of these events is not clear. Regarding the role of NaR, high concentrations of this metabolite do not inhibit WD by itself (Alexandris et al., 2022; Sasaki et al., 2021) and while NaMN may inhibit purified SARM1 (Sasaki et al., 2021), the NaMN/NMN ratio does not predict SARM1 activity like the NAD^+^/NMN ratio does in axons (Alexandris et al., 2022). Nicotinamide adenine dinucleotide phosphate (NADP) has also been suggested as a potent inhibitor of SARM1 (Angeletti et al., 2022), but the physiological role of this inhibition for axons remains unknown.

## SARM1 Activation in WD: Hijacking of NAD^+^ Metabolism

The combined roles of SARM1 as a NAD^+^ hydrolase, “sensor” of NAD^+^ metabolism, and driver of WD, inexorably tie the fate of the axon to its intrinsic NAD^+^ metabolism. This idea explains how loss of NMNAT2 activity after axonal injury, by altering the NAD^+^/NMN ratio and allosterically modifying SARM1, triggers WD. The fact that SARM1 degrades its own inhibitor suggests that SARM1 activation may lead to a potentially catastrophic cascade of events: local depletion of NAD^+^ by SARM1 further reduces the NAD^+^/NMN ratio, leading to further disinhibition of SARM1 and additional NAD^+^ depletion in a quasi-perpetual state of SARM1 activation.

However, dissecting the contributions of NMNAT2 loss and SARM1 activation to axonal NAD^+^ metabolism and understanding the role of these events in axonal demise are not as simple as it may follow from the previous sections.

In healthy neurons, SARM1 activity is minimal and does not contribute significantly to NAD^+^ metabolism (Sasaki et al., 2020). However, the consequences of SARM1 activation are quite dramatic: as mentioned earlier, forced activation of the SARM1-TIR domains *in vitro* is sufficient to deplete neuronal NAD^+^ levels by 90% within 90 min (Gerdts et al., 2015). In this scenario, loss of NAD^+^ is followed by loss of ATP, axonal degeneration, and neuronal cell death (Gerdts et al., 2015). The central role of endogenous SARM1 in driving NAD^+^ depletion after injury can also be demonstrated by comparing NAD^+^ metabolism in transected wt and *Sarm1* KO axons. In wt axons, NMN levels rise by fourfold within 4–6 h of axotomy, and NAD^+^ levels drop by more than fivefold. At the same time, levels of cADPR, serving as a marker of SARM1 activation in neurons (Sasaki et al., 2020), rise by 5- to 10-fold (Alexandris et al., 2022; Sasaki et al., 2020).

By contrast, in transected *Sarm1* KO axons, NAD^+^ levels are largely unchanged despite loss of NMNAT2, and cADPR is nearly undetectable (Sasaki et al., 2020). The role of SARM1 in driving NAD^+^ depletion after injury has also been supported in studies that track the degradation and synthesis of NAD^+^ after injury using labeled metabolites (Sasaki et al., 2016). These studies have demonstrated that NAD^+^ depletion after axonal injury is due to a major increase in NAD^+^ degradation rather than the eventual loss of NAD^+^ synthesis (Sasaki et al., 2016). This disproportionate effect of SARM1 on NAD^+^ levels compared with that of NMNAT2 loss may be explained by the positive feedback mechanism of its activation.

The above findings also allow us to understand the discrete roles of NMNAT2 in axonal maintenance and in WD. While loss of NMNAT2 is deleterious in the presence of SARM1, it is not so in its absence. Ablation of *Sarm1* fully rescues the effects of *Nmnat2* KO in both *in vitro* and an *in vivo* paradigms, and double *Nmnat2/Sarm1* KO neurons and axons seem to maintain NAD^+^ at levels close to their wt counterparts (Gilley et al., 2015). This observation suggests that, in the absence of NAD^+^ degradation by SARM1, NAD^+^ synthesis may be preserved in axons deficient of NMNAT2 without obvious consequences (Gilley et al., 2015). One possibility is that NAD^+^ synthesis is sustained under these conditions because of the involvement of the mitochondrial isoform NMNAT3 (Gilley and Coleman, 2010).

Another important aspect of the relationship between NAD^+^ metabolism and WD is the fact that WD signaling and axonal degeneration are not related to levels of axonal NAD^+^ *per se*. Uninjured axons can remain viable even in the presence of severe NAD^+^ deficiency. Manipulations that reduce NAD^+^ levels by >90% without lowering the NAD^+^/NMN ratio (*e.g*., NAMPT inhibition) do not lead to axonal degeneration (Di Stefano et al., 2015; Sasaki et al., 2020; Sasaki et al., 2016). In this scenario, axons may be able to adapt to the reduced rate of NAD^+^ synthesis by proportionally decreasing NAD^+^ degradation and achieving a new steady state (Sasaki et al., 2016). On the contrary, activation of SARM1 is able to overwhelm any adaptive mechanisms in NAD^+^ metabolism and lead to axonal demise.

## From SARM1 to WD: Downstream Effectors

An important question is whether axonal degeneration is executed by degradation of NAD^+^ *per se*, or by the products of SARM1 enzymatic activity such as free or cyclical ADPR and ADPRP or nicotinic acid adenine dinucleotide phosphate (NaADP) (Angeletti et al., 2022) (Fig. 7). In support of the former, the effect of SARM1-TIR dimerization on axon degeneration *in vitro* may be annulled by strategies that boost NAD^+^ biosynthesis (Gerdts et al., 2015).

Similarly, forced activation of a NADase other than SARM1 with PARP activity has the same effect as SARM1-TIR dimerization, indicating that NAD^+^ degradation is sufficient to cause degeneration unrelated to downstream products (Gerdts et al., 2015). Of note, these experiments involve extraordinary interventions resulting in rapid and catastrophic loss of NAD^+^ levels. However, and in indirect support of an important role of NAD^+^, preventing the injury-induced accumulation of cADPR with a cADPR phosphohydrolase does not prevent axonal degeneration after axotomy (Aksoy et al., 2006; Sasaki et al., 2020).

Furthermore, small doses of mitochondrial toxins can induce SARM1-mediated synthesis of cADPR at levels similar to those observed with axotomy, but do not lead to axonal degeneration (Sasaki et al., 2020). One mechanism by which NAD^+^ depletion may lead to WD is altered Ca^+^ homeostasis. The rapid degradation of NAD^+^ may impair both glycolysis and oxidative phosphorylation, resulting in the loss of mitochondrial potential, generation of reactive oxygen species (ROS), and loss of ATP (Ko et al., 2021). The resulting bioenergetic failure may impair ATP-dependent ionic pumps or ROS-mediated sensitization of transient receptor potential cation channel vanilloid 1 (Kievit et al., 2022; Ko et al., 2021) and lead to a final wave of Ca^2+^ influx, calpain proteolysis, loss of membrane integrity, and axonal fragmentation (Ko et al., 2021).

On the contrary, one cannot exclude a potential additional role for the various enzymatic products of SARM1, particularly cADPR, ADPR, and NaADP, all of which are known calcium mobilizers. Cyclical adenosine diphosphate ribose modulates calcium release through ryanodine receptor (RyR) channels (Ogunbayo et al., 2011). Adenosine diphosphate ribose is an activator of the transient receptor potential cation channel subfamily M member 2 (TRPM2) that enables the influx of Ca^2+^ from the extracellular space (Tóth et al., 2015), and NaADP is one of the most potent Ca^2+^ mobilizers although its target is not clear (Galione, 2019). It was recently shown that antagonism of cADPR signaling by 8-Br–cADPR or knockdown of TRPM2 or RyR can partially rescue axons in the slow, SARM1-dependent, paclitaxel-induced axonal fragmentation (Li et al., 2022). On the contrary, 8-Br–cADPR does not delay the much faster axonal degeneration after axotomy (Li et al., 2022).

## Expanding the Range of WD Signaling Centered on NAD^+^: The Role of c-JUN N-Terminal Kinase Mitogen-Activated Protein Kinase Cascade

In the previous sections, we have discussed how loss of NMNAT2 and SARM1 signaling hijacks axonal NAD^+^ metabolism to trigger axonal degeneration. But what regulates NMNAT2 levels in the axon and what causes NMNAT2 loss after injury? All evidence shows that levels of NMNAT2 are determined by a balance between anterograde transport plus probable local translation and its local rapid degradation. NMNAT2 undergoes bidirectional fast axonal transport in vesicles (Milde et al., 2013) and, thus, loss of NMNAT2 due to disruption of axonal transport may lead to WD in diverse conditions associated with axon transport deficits.

The latter include biomechanical disruption in the course of traumatic brain injury (Koliatsos and Alexandris, 2019), mutations in axon transport-related genes in some hereditary neuropathies (Beijer et al., 2019), microtubule toxicity associated with chemotherapeutic agents (Fukuda et al., 2017), and proteinopathic or other unknown conditions associated with neurodegenerative diseases (Milde et al., 2015; Millecamps and Julien, 2013).

On the contrary, NMNAT2 is degraded by two distinct pathways based on its status of palmitoylation, a post-translational modification that tethers NMNAT2 to membranes (Summers et al., 2018). The palmitoylated majority is targeted for degradation *via* the c-JUN N-terminal kinase (JNK) mitogen-activated protein kinase (MAPK) pathway driven by the MAP3Ks dual leucine zipper kinase (DLK) and leucine zipper kinase (LZK) (Summers et al., 2018). The less abundant nonpalmitoylated fraction is degraded by the Phr1/Skp1a/Fbxo45 ligase complex (Summers et al., 2018). Inactivation of both pathways leads to synergistic accumulation of NMNAT2 and significantly delays axonal degeneration after axotomy (Summers et al., 2018), while DLK inhibition up to 2 h postinjury ameliorates loss of NMNAT2, attenuates SARM1 activation, and delays WD (Alexandris et al., 2022).

The JNK MAPK (also referred to as DLK-JNK) signaling cascade belongs to a family of highly conserved pathways activated in response to oxidative stress, inflammatory cytokines, mitogens, growth factors, and activation of various G-protein–coupled receptors (Morrison, 2012; Zeke et al., 2016). By virtue of its role in modulating NMNAT2 levels, axonal MAPK signaling may lower the threshold for or instruct WD in response to local stressors. For example, DLK-JNK cascade activation plays a key part in SARM1-dependent degeneration after neurotrophic withdrawal (Gerdts et al., 2013) or biomechanical injury (Alexandris et al., 2022; Gerdts et al., 2013; Yang et al., 2015). The DLK-JNK cascade may also be stimulated downstream to SARM1 activation (Yang et al., 2015), thus bearing the potential of triggering a feed-forward mechanism. This possibility is also supported by evidence of direct phosphorylation of SARM1 by JNK resulting in increased enzymatic activity (Murata et al., 2018).

Depletion of NMNAT2 and WD may also occur secondary to mitochondrial dysfunction, as in the case of mitochondrial toxins (Loreto et al., 2020; Summers et al., 2014). In this case, ROS generation may be a necessary upstream step (Press and Milbrandt, 2008), although the mechanism linking excess ROS and NMNAT2 depletion has not been established. A feed-forward mechanism between mitochondrial dysfunction and SARM1 activation is also possible, because SARM1 activation results in mitochondrial depolarization and loss of motility after axotomy (Ko et al., 2021).

**FIG. 8.**
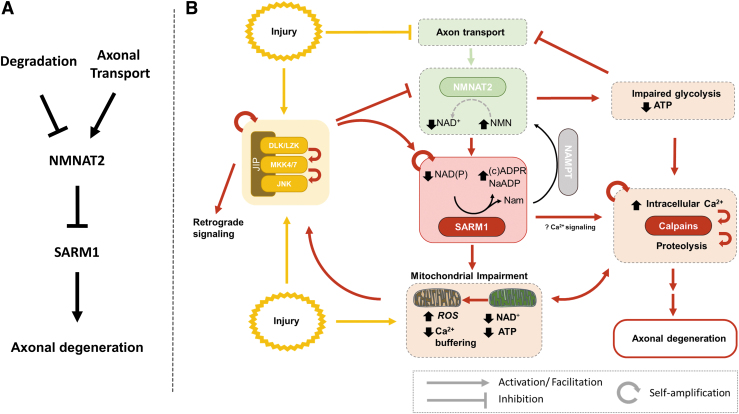
**WD signaling pathways. (A)** Core players are SARM1 as the instructor and executor of WD and NMNAT2 as an indirect suppressor of SARM1 activity. NMNAT2 levels are regulated by a balance between axonal delivery and degradation. **(B)** WD signaling (*red arrows*) can be initiated by different types of axonal perturbations (*yellow arrows*) that result in loss of NMNAT2 activity. This can be achieved *via* direct injury to the axon and loss of anterograde axon transport, and/or increased degradation of NMNAT2 secondary to increased activity of the DLK-JNK cascade (*yellow box*). The DLK-JNK cascade can be activated by different abiotic and biotic stressors (*yellow arrows*), including direct axonal injury and probably mitochondrial impairment, and can be self-amplified. Loss of NMNAT2 activity results in accumulation of NMN, reduced NAD^+^ synthesis, and allosteric activation of SARM1 due to a decrease in the NAD^+^/NMN ratio. SARM1 activation results in local NAD^+^ degradation in a feed-forward manner and generation of Ca^2+^ mobilizers. JNK-mediated SARM1 phosphorylation may further increase its activity. Severe NAD^+^ depletion may also lead to mitochondrial depolarization and bioenergetic failure, and loss of both cytosolic and mitochondrial ATP synthesis; as a consequence, intracellular Ca^2+^ homeostasis cannot be maintained, resulting in calpain activation, proteolysis of axonal proteins, and axonal degeneration. DLK, dual leucine zipper kinase; JNK, c-JUN N-terminal kinase.

As noted previously, WD-related signaling appears to be featured by several self-amplifying steps, including the feed-forward activation between the DLK-JNK cascade (Niu et al., 2022) and SARM1, as well as the bidirectional relationship between SARM1 activation and mitochondrial dysfunction. A more detailed model showing some of these features is presented in Figure 8. Positive feedback mechanisms are common in biological processes leading to irreversible cellular changes (Mitrophanov and Groisman, 2008), and also explain how local axonal injury, for example, after transection, may lead to relatively synchronous rapid fragmentation across the length of the axon after a certain induction period. On the contrary, such mechanisms also raise the question as to how neurons may safeguard against inadvertent SARM1 activation. One obvious way is the inherent conformational stability of the enzyme. Based on cell-free experiments exploring the activation of human SARM1 by NMN, the conformational change resulting in enzymatic activation occurs rather slowly, that is, in the scale of hours (Figley et al., 2021). If this is also the case *in vivo*, such inherent stability may act as a fail-safe against accidental activation of SARM1.

## Additional Insights on WD Signaling from Studies in *D. melanogaster*

While in this review we have focused primarily on the characterization of NAD^+^ metabolism and WD signaling in mammalian neurons, parallel work in Drosophila has emphasized the evolutionarily conserved regulation of these mechanisms and has also generated some novel insights. In Drosophila NMN and NAD^+^ synthesis progresses through an NRK homolog (dNrk) instead of NAMPT and there is only one NMNAT homolog (dNmnat), Yet WD is also instructed by the loss of dNmnat, accumulation of NMN, and the allosteric activation of dSarm (Llobet Rosell et al., 2022). Ablation or knockdown of dNmnat leads to rapid axonal degeneration (Fang et al., 2012). Conversely, suppression of dNmnat degradation by ablation of the E3 ubiquitin ligase Highwire (Hiw), suppression of NMN accumulation by the ectopic expression of a bacterial NMN deamidase, and loss-of-function mutations of dSarm, all afford long-lasting axonal and synaptic protection for weeks or months (Fang et al., 2012; Llobet Rosell et al., 2022; Neukomm et al., 2014; Osterloh et al., 2012; Xiong et al., 2012). Importantly, in several of these scenarios, axons remain functionally intact.

A genetic screen for modulators of WD in Drosophila has contributed another piece of the WD puzzle, the discovery of the BTB/BACK domain protein Axundead (Axed) (Neukomm et al., 2017). Deletion of Axundead was reported to prevent axonal degeneration in diverse scenarios, including axotomy, the loss of dNmnat, and the expression of a constitutively active dSarm variant, suggesting that it may act downstream of SARM1 (Neukomm et al., 2017). However, its mechanism of action is still unclear, and the role of four mammalian paralogues of Axundead in WD remains uncertain.

## WD Signaling in Disease: From Traumatic Axonal Injury to Neurodegeneration

The recognition that axonal degeneration is not the passive outcome of axonal injury but the result of activation of highly conserved molecular programs centered on Wld^S^ and SARM1 offers the opportunity of a mechanistic understanding of axonopathies and possible therapeutic targeting of key molecular steps. This is especially so because, in most disease states, axonal injury is not as complete as in axotomy, and perturbed axons may be rescuable before the triggering of WD (Koliatsos and Alexandris, 2019).

For example, *in vivo* imaging of axons in a model of experimental autoimmune encephalomyelitis (EAE) has revealed that swollen injured axons can persist for several days before progressing to degeneration or recovery (Nikic et al., 2011). A prolonged postinjury state of survival has been also demonstrated in an *in vitro* model of axonal exposure to rotenone (Hughes et al., 2021); in this model, pharmacological inhibition of SARM1 after the injury can prevent degeneration and allow axons to recover (Hughes et al., 2021).

The realization that WD is a molecular program akin to programmed cell death and the related opportunities for understanding pathogenesis and designing novel therapeutics have led to a broader consideration of WD mechanisms beyond the classical axotomy scenario. It now appears that interference with WD protects against axonal degeneration in several disease models associated with axonopathy, including traumatic brain injury (Alexandris et al., 2023a; Alexandris et al., 2023b; Bradshaw et al., 2021; Henninger et al., 2016; Marion et al., 2019; Maynard et al., 2020; Ziogas and Koliatsos, 2018), stroke (Gillingwater et al., 2004), EAE (Kaneko et al., 2006; Viar et al., 2020), neurotoxic parkinsonism (Hasbani and O'Malley, 2006; Sajadi et al., 2004), diabetic and chemotherapy-induced neuropathy (CIPN) (Geisler et al., 2016; Turkiew et al., 2017; Wang et al., 2002), retinal ischemia and glaucoma (Beirowski et al., 2008; Howell et al., 2013; Zhu et al., 2013), and neuroinflammatory conditions (Ko et al., 2020).

Besides establishing a broader role of NAD^+^-related metabolic pathways in disease, these studies have offered a more subtle understanding of the role of WD signaling in axonal pathology. For example, expression of Wld^S^ protects against nigrostriatal axonal degeneration when axons are lesioned with 6-hydroxydopamine in the medial forebrain bundle but not in their terminals (Sajadi et al., 2004). Similarly, *Sarm1* ablation strongly protects the distal segments of axons after traumatic axonal injury but does not protect proximal axons or cell bodies (Alexandris et al., 2023a). In addition, the robust axonal protection observed in the first 1 or 2 weeks with inhibition of WD may not always translate in long-term protection, at least to the same degree (Alexandris et al., 2023a; Viar et al., 2020).

**FIG. 9.**
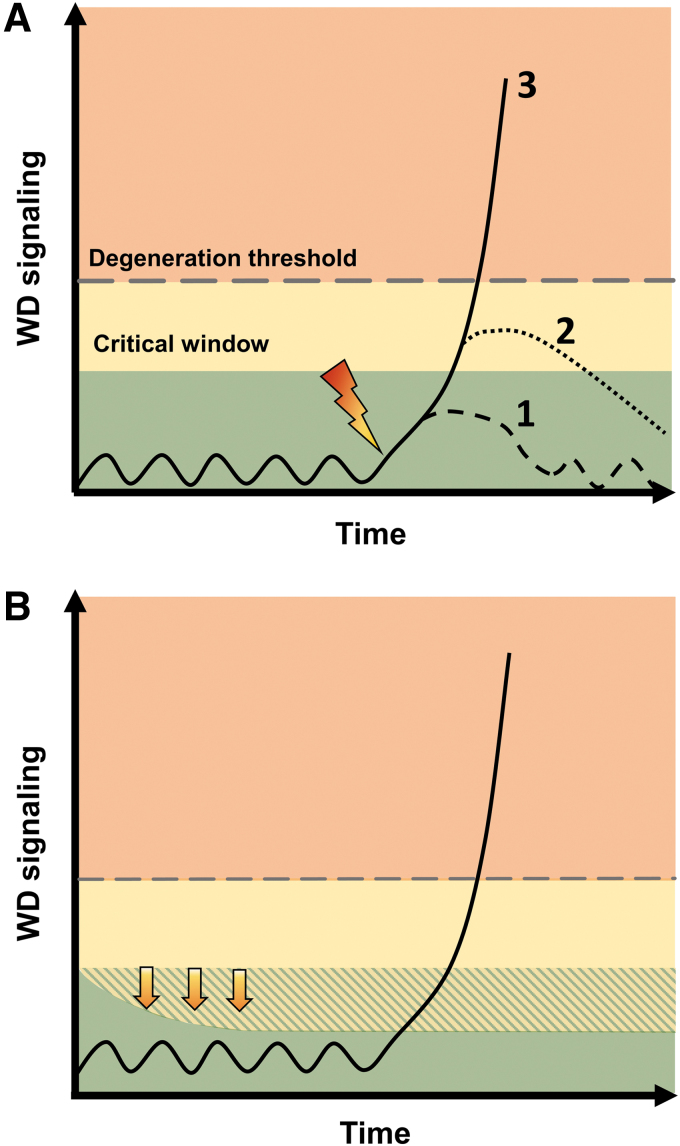
**Models of axonal injury and degeneration related to WD signaling. (A)** A healthy axon is subjected to an acute or subacute axonal insult (*lightening strike*). The axon is natively resistant to minor insults and the transient turn on of WD signaling, principally activation of SARM1 (*line 1*), but more severe insults that drive SARM1 activity to critical levels (*lines 2* and *3*) trigger feed-forward cascades and eventually axon degeneration (also see Fig. 8). Inhibition of SARM1 within a critical window may abort progression of WD and allow the axon to recover (*line 2*). **(B)** The threshold that triggers WD depends on several factors including the availability and turnover of NMNAT. Change in threshold, for example, due to transient or chronic deficits in NMNAT2 activity or NAD^+^ metabolism (*arrows*) may predispose axons to WD with future mild insults or small variations in SARM1 activity.

So far, our understanding of WD and its contribution to disease has been primarily based on models that encompass acute or subacute axonal perturbations in otherwise healthy neurons. In this context, as shown in the model presented in Figure 9, the axon may be resistant to small perturbations associated with transient activation of SARM1 (line 1), but more severe insults that drive SARM1 activity above a certain threshold (lines 2 and 3) may commit axons to degeneration. In acute injury, inhibition of SARM1 within a critical window may prevent triggering of WD and allow the axon to recover (line 2). On the contrary, axonal protection by Wld^S^ or *Sarm1* KO has also been reported in chronic or progressive conditions such as chronic glaucoma (Howell et al., 2013), motor neuronopathy (Ferri et al., 2003), and in TDP-43–linked motor neuron degeneration (White et al., 2019). In these chronic scenarios, WD may be triggered any time by the same mechanisms as in acute injury. Alternatively, the underlying process may just lower the triggering threshold.

The latter may be determined by levels of NMNAT2: stimulation of the DLK-JNK cascade in axons that leads to NMNAT2 degradation is not always sufficient to induce degeneration, but can lower the threshold for degeneration after a second insult (Summers et al., 2020). A potential prodegenerative role of chronic SARM1 activation has also been suggested in some cases of amyotrophic lateral sclerosis (ALS) featured by rare SARM1 variants that confer constitutive enzymatic activation (Bloom et al., 2022; Gilley et al., 2021). The idea of SARM1 contributing to chronic neurodegeneration by a feed-forward mechanism centered on mitochondrial pathology has been recently proposed in Charcot-Marie-Tooth disease type 2A caused by mutations in the mitochondrial gene mitofusin 2 (MFN2) (Sato-Yamada et al., 2022).

A brief comment is in order regarding the early and disproportional axonal degeneration observed in sporadic neurodegenerative diseases (Adalbert and Coleman, 2013; Alexandris et al., 2020; Dadon-Nachum et al., 2011; Tagliaferro and Burke, 2016). In this case, establishing a causal role of WD signaling and axonal NAD^+^ metabolism is more challenging. Although there is some indirect evidence implicating WD-related pathways such as reductions in NMNAT2 expression (Ali et al., 2017), altered JNK MAPK signaling (de Los Reyes Corrales et al., 2021), and altered NAD^+^ metabolism (Fang et al., 2017), there is clear need for confirmatory evidence.

## Roles of Axonal NAD^+^ Metabolism Beyond WD

The previous sections have argued for a central role of metabolic pathways converging on NAD^+^ in the regulation of WD. Here, we briefly discuss the implication of axonal NAD^+^ metabolism in other aspects of axonal physiology and metabolism that are less well understood. As explained in the Essentials of NAD^+^ Metabolism with Emphasis on Mammalian Neurons section, NAD^+^ serves as a cofactor in reductive–oxidative metabolism, and is also a substrate for enzymes such as sirtuins and PARPs that have distinct regulatory functions and may contribute to axonal viability. Several of these enzymes are expressed in axons (Chuang et al., 2018; Estrada-Bernal et al., 2012; Shigeoka et al., 2016) and may support NAD^+^-dependent axonal functions. In contrast to WD signaling that hijacks NAD^+^ metabolism to instruct axonal degeneration and is not directly related to NAD^+^ levels, the function of most sirtuins and PARPs is directly linked to NAD^+^ availability (Imai and Guarente, 2016) and may be more susceptible to NAD^+^ deficits in the short- or long term.

Sirtuin 2 is the main cytoplasmic protein of the sirtuin family (Wu et al., 2022). It is a predominantly neuronal and oligodendrocytic protein and, in neurons, it is found in both the soma perikaryon and axon (Maxwell et al., 2011). This sirtuin contributes to the modulation of cytoskeletal dynamics (Suzuki and Koike, 2007), and can regulate microtubule-dependent transport and autophagic flux in neurons (Esteves et al., 2019). Sirtuin 2 activity may inhibit axon growth by impairing cytoskeletal growth cone dynamics during development, but may be required for mature neuronal function because its ablation may cause axonal pathology and age-associated movement deficits (Fourcade et al., 2017).

The mitochondrial sirtuins SIRT3 and SIRT5 may also be important for axonal viability, primarily due to their role in maintaining mitochondrial integrity (Wu et al., 2022). Specifically, mitochondrial function can be negatively impacted by the predominantly nonenzymatic acylation of their proteins (Hong et al., 2016). The NAD^+^-dependent deacylation of mitochondrial proteins by SIRT3 (and maybe SIRT5) restores protein function, and represents an important repair mechanism (Wagner and Payne, 2013; Weinert et al., 2015). For instance, SIRT3 is required for the deacetylation of ETC proteins to restore mitochondrial respiration (Ahn et al., 2008), and of SOD2 and IDH2 to support antioxidant defense (Qiu et al., 2010; Yu et al., 2012). Sirtuin 3 also regulates mitochondrial transcription to support oxidative phosphorylation (Liu et al., 2014) and the mitochondrial unfolded protein response (Papa and Germain, 2014), and can suppress caspase-dependent axonal degeneration (Magnifico et al., 2013). Conversely, depletion of mitochondrial NAD^+^ levels in neurons leads to increased mitochondrial protein acetylation, high ROS production, and excessive mitochondrial fragmentation, all of which can be ameliorated by stimulation of SIRT3 activity (Klimova et al., 2020). Sirtuin 5 has been much less studied, but its ablation has been associated with increased neuronal degeneration after neurotoxic insults (Li and Liu, 2016; Liu et al., 2015; Xiao et al., 2021), probably due to its role in suppressing ROS, in supporting ATP synthesis, and in promoting autophagy (Wu et al., 2021; Xiao et al., 2021). Therefore, reduction in expression levels of SIRT3 and SIRT5 or impaired activity due to NAD^+^ deficiency may increase neuronal vulnerability in disease (Liu et al., 2015; Wu et al., 2021; Xiao et al., 2021; Yin et al., 2018).

While SIRT1 and PARP1 are predominantly nuclear NAD^+^-consuming enzymes, they may also be localized in axons where they serve axon-specific roles. SIRT1 is found in the growth cone, and may play a role in promoting axonal elongation and branching (Li et al., 2013). In a similar vein, while the extranuclear role of PARP1 has been debated, PARP1 transcripts have been isolated from axonal growth cones, and the protein has been isolated from the axonal proteome of primary cortical neurons (Chuang et al., 2018; Poulopoulos et al., 2019). Axonal PARP1 may mediate the effects of extrinsic growth-inhibitory signals (Brochier et al., 2015), although its role in central nervous system regeneration has been debated (Wang et al., 2016).

## NAD^+^ Metabolism as Therapeutic Target for Axonopathies

NAD^+^ metabolism is a key factor in axonal maintenance, but may also be opportunistically utilized to drive axonal neurodegeneration in neurological diseases and their models. For this reason, the pharmacological modulation of NAD^+^ metabolism presents a great therapeutic opportunity.

The clinical potential of suppressing WD-related NAD^+^ metabolism is further supported by a clinically promising therapeutic window: *in vitro* models indicate that axonal degeneration can be successfully suppressed with manipulation of NAD^+^-related signals for up to 8 h after injury (Alexandris et al., 2022). *In vivo*, the time interval to WD trigger is much slower, for example, 36 h for the mouse sciatic nerve (Beirowski et al., 2004), suggesting the possibility of an even wider therapeutic window in clinically relevant settings.

There are several potential strategies for therapeutic modulation of NAD^+^ metabolism. In axonopathies that are driven at least partially by SARM1 activation, the use of SARM1 inhibitors is the most straightforward approach. The race for the discovery and validation of SARM1 inhibitors is already underway, and results from preclinical models appear promising (Bosanac et al., 2021; Bratkowski et al., 2022; Feldman et al., 2022; Hughes et al., 2021). An alternative strategy is indirect inhibition *via* the modulation of NAD^+^ metabolism. Liu et al. (2018a) discovered that neurons have the capacity to synthesize NAD^+^ from NaR *via* the deamidated salvage pathway, which bypasses NMN synthesis, and can potentiate the protective effects of NAMPT inhibitors in vincristine-induced axonal degeneration. We further showed that the combination of NAMPT-inhibition with NaR supplementation suppresses SARM1 activity by improving the NAD^+^/NMN ratio, and that this treatment, in turn, can robustly suppress WD for several days (Alexandris et al., 2022). Therefore, subverting neuronal metabolism from the Nam salvage to the deamidated route may be a promising approach, particularly due to the availability of NAMPT inhibitors such as Daporinad (FK866) that have been already clinically tested for other indications (Holen et al., 2008).

Another upstream approach is to suppress the JNK MAPK-dependent loss of NMNAT2, for example, with the use of DLK inhibitors (Alexandris et al., 2022). This intervention has the added benefits of reducing prodegenerative signaling in the cell bodies (Welsbie et al., 2019), and of possibly breaking feed-forward WD signaling loops linking SARM1 and the JNK MAPK cascade. Stimulating NMNAT2 synthesis is another version of this strategy. For example, a screen for modulators of NMNAT2 expression in cortical neurons identified caffeine as a positive modulator of *Nmnat2*, and short-term treatment with caffeine was able to restore NMNAT2 expression in the rTg4510 tauopathy mouse model (Ali et al., 2017).

The augmentation of NAD^+^ biosynthesis with supplementation of NAD^+^ precursors is also a highly active area of research (Radenkovic et al., 2020; Reiten et al., 2021). For instance, increasing NAD^+^ synthesis with Nam has shown benefits in both preclinical (Williams et al., 2017, 2018) and clinical trials in glaucoma (De Moraes et al., 2022; Hui et al., 2020), while prophylactic supplementation of NAD^+^ precursors prevented the development of neuropathy in rodent models of diabetes (Chandrasekaran et al., 2022).

Similarly, the use of NAMPT activators has been proposed as a strategy to stimulate the Nam core recycling pathway, and may ameliorate experimental CIPN when used for pre-exposure prophylaxis (Wang et al., 2022). Currently, >30 of 600 clinical trials involving NAD^+^ precursors are targeting neurological conditions such as peripheral neuropathy, glaucoma, Alzheimer's disease, Parkinson's disease, and ALS (clinicaltrials.gov). However, given the highly compartmentalized nature of NAD^+^ metabolism, it is still not clear how different NAD^+^ precursors may affect axonal NAD^+^ metabolism *in vivo* and, more importantly, improve the NAD^+^/NMN ratio in already compromised axons. This is due to the fact that, when NMNAT2 activity is reduced, supplementation of NAD^+^ precursors or activation of NAMPT may increase intra-axonal NMN and lead to SARM1 activation.

To the point, NR supplementation has the potential to increase NMN levels and activate SARM1 even in uninjured axons, and as a result can accelerate axonal degeneration (Figley et al., 2021). In addition, NAD^+^ precursors when taken orally can be metabolized by the enteric microbiome (Chellappa et al., 2022), intestinal tissues and liver, and transformed to other precursors before arriving to target tissues, whereas the blood–brain barrier may restrict which species may enter the brain (Liu et al., 2018b). Therefore, the option of using NAD^+^ precursors in axonopathies presents pharmacokinetic and pharmacodynamic challenges, and should be viewed with caution. This is especially so as “NAD^+^ boosting” has become a very popular claim in over-the-counter supplements.

In conclusion, clarifying the relationship between NAD^+^ metabolism and WD signaling is extremely important. The central role of NAD^+^ in axonal viability and degeneration is evident not only from findings in a variety of animal models but also the re-examination of the sparse older literature on pellagra. The growing understanding of WD at the molecular level and the formulation of relevant molecular targets have great potential for the treatment of axonopathies. On the other hand, the role of NAD^+^ metabolism in regulating WD is complex and may be distinct from its role in maintenance intact axons. Delving further into the mechanisms by which NAD^+^ contributes to axonal viability and into the complex relationships between NAD^+^ metabolism and WD signaling will be important for the development of effective strategies that not only suppress axonal degeneration but also promote axonal health.

## Abbreviations Used

ADPR: ADP-ribose
ALS: amyotrophic lateral sclerosis
ARM: armadillo repeat
ARTC: ADP-ribosyltransferase C2/C3 toxin-like
cADPR: cyclical ADP-ribose
CIPN: chemotherapy-induced neuropathy
DLK: dual leucine zipper kinase
DRG: dorsal root ganglion
EAE: experimental autoimmune encephalomyelitis
eNAMPT: extracellular nicotinamide phosphoribosyltransferase
ETC: electron transport chain
iNAMPT: intracellular nicotinamide phosphoribosyltransferase
JNK: c-JUN N-terminal kinase
MAPK: mitogen-activated protein kinase
MNA: 1-methylnicotinamide
MyD88: myeloid differentiation primary response gene 88
Na: nicotinic acid
NaAD: nicotinic acid adenine dinucleotide
NaADP: nicotinic acid adenine dinucleotide phosphate
NAD^+^: nicotinamide adenine dinucleotide (oxidized)
NADH: nicotinamide adenine dinucleotide (reduced)
NADSYN: NAD^+^ synthase
Nam: nicotinamide
NaMN: nicotinic acid mononucleotide
NAMPT: nicotinamide phosphoribosyltransferase
NAPRT: nicotinate phosphoribosyltransferase
NaR: nicotinic acid riboside
NMN: nicotinamide mononucleotide
NMNAT: nicotinamide mononucleotide adenylyltransferase
NNMT: nicotinamide n-methyltransferase
NPP1: nucleotide pyrophosphatase/phosphodiesterase 1
NR: nicotinamide riboside
NRK: nicotinamide riboside kinase
PARP: poly-ADPribosylatransferase
PNP: purine nucleoside phosphorylase
QA: quinolinic acid
QPRT: quinolinate phosphoribosyl transferase
ROS: reactive oxygen species
RyR: ryanodine receptor
SAM: sterile alpha motif
SARM1: sterile alpha and TIR motif containing 1
TIR: toll-interleukin-1 receptor
TRPM2: transient receptor potential cation channel subfamily M member 2
WD: Wallerian degeneration
Wld^S^: Wallerian degeneration slow
